# Optimized mobilization of MHC class I- and II- restricted immunity by dendritic cell vaccine potentiates cancer therapy

**DOI:** 10.7150/thno.71760

**Published:** 2022-04-24

**Authors:** Yingying Shi, Yu Liu, Jiaxin Huang, Zhenyu Luo, Xuemeng Guo, Mengshi Jiang, Xiang Li, Yichao Lu, Xu Liu, Xinyu Shan, Lihua Luo, Jian You

**Affiliations:** College of Pharmaceutical Sciences, Zhejiang University, 866 Yuhangtang Road, Hangzhou, Zhejiang 310058, P. R. China.

**Keywords:** MHC-restricted immunity, cationic nanoemulsions, mRNA-DCs, protein-DCs, cancer therapy

## Abstract

**Background:** The participation of major histocompatibility complex (MHC) in antigen presentation shapes both the breadth and magnitude of specific T cell response. Dendritic cells (DCs) activated with nucleic acid or protein that encodes/incorporates multiple antigenic epitopes elicit MHC class I- and II- biased immunity, respectively. Studies demonstrate that an elevated MHC class I-directed CD8^+^ cytotoxicity T lymphocyte (CTL) response is able to provide survival benefits to patient with malignant tumor. However, a fully effective cancer therapy must elicit a diverse repertoire of both CD4^+^ and CD8^+^ T cell responses, raising demands on a multifaceted activation of the MHC system. Current therapeutic strategies usually lack an orchestrated mobilization of the MHC class I and II responses. Vaccines with little synergistic effect or unmanageable elicitation of the CD4^+^ and CD8^+^ T cell immunity usually fail to induce a potent and durable anti-tumor protection.

**Methods:** Here, cationic nanoemulsions (CNEs) complexed with full-length tumor model antigen ovalbumin (OVA) in the form of mRNA or protein were constructed and used as two antigenic platforms to prepare DCs vaccines with tailored MHC participation (i.e., mRNA-DCs and protein-DCs). In exploring a vaccine regimen with optimal tumor suppressing effect, the mixing ratio of mRNA-DCs and protein-DCs was manipulated.

**Results:** Therapeutic DCs vaccines involving both antigenic platforms induced better anti-tumor immunity in murine E.G7-OVA lymphoma model and B16-OVA melanoma model, which can be further augmented upon a meticulous reallocation of the MHC class I and II responses.

**Conclusion:** This work indicated that a simultaneous and coordinated mobilization of the MHC-restricted immunity might potentiate cancer therapy.

## Introduction

Dendritic cells (DCs) are the key mediators of antigen presentation, during which the participation of major histocompatibility complex (MHC) largely determines the type and magnitude of ensuing T cell response, shaping specific immunity against tumor and infection. The presentation fate of antigen is primarily subject to several intrinsic factors, including the sequential/structural characteristics and the spatiotemporal distribution of antigen 1-3. Generally, endogenously synthesized antigens are degraded into peptide fragments and favorably assembled with MHC class I molecules to form peptide-MHC class I complexes (p-MHC I) that activate specific CD8^+^ T cells, while exogenously endocytosed antigens tend to form p-MHC II that prime CD4^+^ T helper (Th) cells.

Several lines of evidence suggest that amplifying MHC class I-restricted immunity by DCs restrains the initiation, progression and metastasis of tumor 4, 5. There are two main strategies for up-regulating MHC class I response, namely, facilitating the cross presentation of exogenous protein/peptide antigens 2 and adopting nucleic acid-based systems for prioritized endogenous antigen expression. The former includes: 1) promoting the cytosolic distribution of antigen for cytoplasmic proteasome-mediated degradation that favors the generation of MHC class I-restricted epitopes 6, 7; 2) appropriate alkalization of endosome/lysosome acidity to avoid the over-degradation of antigen and maintain the structural integrity of MHC class I epitopes 8-10; 3) targeted delivery of antigen to specific tissue (lymph nodes) 11-13, cell type (DCs, especially conventional type 1 DCs (cDC1)) 14-16 and even subcellular organelle (early endosome 17, endoplasmic reticulum 18, 19) that benefits the development of MHC class I-associated antigen process and presentation. On the other hand, the later mainly relies on bio-genetic engineering techniques to construct DNA, mRNA and virus vector that encode a single or multiple tumor-specific epitopes for endogenous expression of antigen and privileged presentation via p-MHC I. Moreover, the supplementation of stimulating cytokines and adjuvants promotes both the quantity and quality of DCs, which may also facilitate the MHC class I-restricted immunity 20, 21.

However, a potent and durable anti-tumor immunoresponse depends heavily on the interplay between CD4^+^ and CD8^+^ T cells, and the co-presence of specific CD4^+^ and CD8^+^ T cells in the tumor tissue is regarded as a good prognostic factor 22, 23, indicating that a concurrent elicitation of the MHC class I and II immunity is needed 24-26. Moreover, it is reported that MHC class II-restricted response is required for augmented immune-mediated elimination of tumors 23, 27; CD4^+^ Th cells help sustain the cytolytic function and promote the memory commitment of CD8^+^ T cells during infection, cancer and immunization 28-30; and MHC class I tumor immunogenicity was essential for triggering tumor-directed CD4^+^ T cells, while tumor-specific CD8^+^ T cell response requires Th1-polarized CD4^+^ Th cells for efficient tumor suppression 31.

A major limitation of current protein- or mRNA- based standalone vaccine strategies is the lack of a multifaceted and coordinated mobilization of the MHC system, which usually leads to a failure in generating compelling anti-tumor clinical effects 32, 33. The focus of this study is to determine whether the anti-neoplastic immune response could be improved by an optimized involvement of the MHC class I and II immunity. Herein, we used mRNA- and protein- based antigenic platforms to prepare DCs vaccines with tunable MHC response and revealed that a fully effective immunotherapy required simultaneous and coordinated elicitation of both MHC class I and II responses.

## Results

### Preparation and characterization of cationic nanoemulsions

Incorporating multiple MHC class I- and II- binding epitopes, full-length or long-peptide tumor associated antigens (TAAs) and/or tumor-specific antigens (TSAs) not only mediate the elimination of tumor via polyclonal immune responses 34, but prevent the relapse of tumor through the establishment of multi-epitope immune memory 35. DCs activated with mRNA or protein encodes/incorporates a diversified repertoire of tumor epitopes elicit MHC class I- and II- biased immunity respectively, which eventually leads to a differential activation preference for CD8^+^ T and CD4^+^ T cells 36. However, naked mRNA can barely penetrate the cell membrane to reach its target of action (cytoplasm) due to an anionic and hydrophilic nature 37, which is further challenged by its high susceptibility to enzymatic degradation. Similarly, protein/antibody-based therapeutics suffer from heterogeneous surface charges, large molecular weights, and fragile tertiary structures that prevent them from entering the cells, impeding their biopharmaceutical applications 38, 39. In these regards, a multifunctional carrier capable of loading both mRNA and protein for efficient intracellular delivery is needed to explore the immunological consequences of different antigenic platforms without introducing additional variables 40.

We previously found that nanoemulsions incorporating vitamin E (VE) displayed good biosafety and long-term stability 41. Here, VE-contained and 1,2-dioleoyl-3-trimethylammonium-propane (DOTAP)-based cationic nanoemulsions (CNEs) were prepared to complex with mRNA via electrostatic interaction, or to load with protein through ionic forces (OVA protein was used as the model antigen, which was negatively charged when dissolved in deionized water, zeta potential: -5.813 ± 0.4136 mV, **Figure S1**) and/or Van der Waals interaction 42, 43, providing a platform for intracellular delivery of functional biomacromolecules.

**Figure 1:**
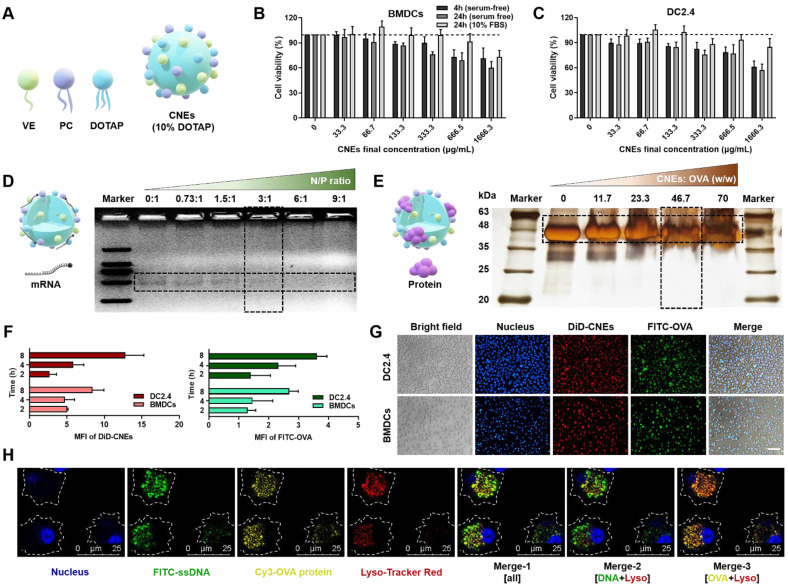
** Characterization of CNEs capable of complexing both mRNA and protein. (A)** Structure diagram of DOTAP-based CNEs. VE: vitamin E, PC: phosphatidylcholine, DOTAP: 1,2-dioleoyl-3-trimethylammonium-propane. **(B-C)** Cell viability of BMDCs (**B**) and DC2.4 (**C**) treated with CNEs-1 (10% DOTAP, w/w) at different concentrations in cell culture medium with or without serum for 4 or 24 h, n = 4.** (D)** Structure illustration of mRNA-CNEs complexes (left), and analysis of mRNA complexation with agarose gel electrophoresis assay (right). 300 ng of eGFP mRNA was incubated with CNEs-1 at a N/P ratio of 0:1, 0.73:1, 1.5:1, 3:1, 6:1 or 9:1. **(E)** Structure illustration of protein-CNEs complexes (left), and determination of the protein loading ability using fast silver stain assay (right). 10 µg of OVA protein was incubated with CNEs-1 at a lipid/protein mass ratio of 0, 11.7, 23.3, 46.7 or 70.** (F-G)** Cellular uptake (**F**, at 2, 4, and 8 h post treatment) and representative fluorescence images (**G**, at 8 h) of protein-CNEs complexes by DC2.4 and BMDCs. Images were analyzed by Image J to semi-quantitate the mean fluorescence intensity (MFI, per cell) of FITC-OVA and DiD-labelled CNEs-1, n = 3. Scale bar: 100 µm. All error bars were expressed as ± SD.** (H)** Confocal microscopic observation of the co-localization of nucleus (blue), lysosome (red), FITC-ssDNA (green) and Cy3-OVA protein (yellow) in BMDCs at 12 h post treatment as rendered by CNEs-1. Scale bar, 25 µm.

Firstly, oil-in-water CNEs containing 10% (w/w) DOTAP (CNEs-1) was prepared by a high-energy emulsification method (**Figure 1A**), which displayed little cytotoxicity to bone marrow-derived DCs (BMDCs, **Figure 1B**) and immortalized DC2.4 cells (**Figure 1C**) in both serum-free and 10% fetal bovine serum (FBS)-supplemented culture medium provided that the final lipid concentration was no more than 133.3 μg/mL. At a N/P ratio of 3 (**Figure 1D**) and a lipid/protein mass ratio of 46.7 (**Figure 1E**), most mRNA and OVA were complexed by CNEs-1, and the resulting complexes were quite stable within 6 h when stored at 4 °C (**Figure S2**). When FITC-conjugated OVA protein was used (green signal) and CNEs were fluorescence labelled with DiD (red signal), the *in vitro* uptake behavior of OVA-loaded CNEs by DC2.4 and BMDCs suggested that a great number of OVA was internalized by cells at 4-8 h (**Figure 1F-G**). Next, we studied the intracellular release of cargos by CNEs in BMDCs using confocal laser scanning microscopy (CLSM). FITC-labelled single strand DNA (FITC-ssDNA, used as the model nucleic acid) and Cy3-labled OVA protein (Cy3-OVA) were concurrently loaded onto CNEs-1 before administration. Results showed that at 12 h post treatment, most ssDNA (green signal) escaped lysosome (red signal) and diffused into the cytoplasm (**Figure 1H**, Merge-2), which was conducive for maintaining their structural integrity and biological activity (generating endogenous protein). Meanwhile, Cy3-OVA (yellow signal) displayed strong co-localization with lysosomes (**Figure 1H**, Merge-3), which might facilitate the development of MHC class II-mediated antigen process and presentation 44, 45.

It should be mentioned that the physicochemical variability of different protein/peptide-based antigens might affect the complexation process and result in unmanageable cargo loading. In this case, modifications that alter the net charge and/or hydropathy of antigens may be needed to facilitate the antigen loading 32, which has been well studied by Qin et al. 38 and Chang et al. 43, and was not investigated in this work.

### mRNA transfection by CNEs in DCs

**Figure 2:**
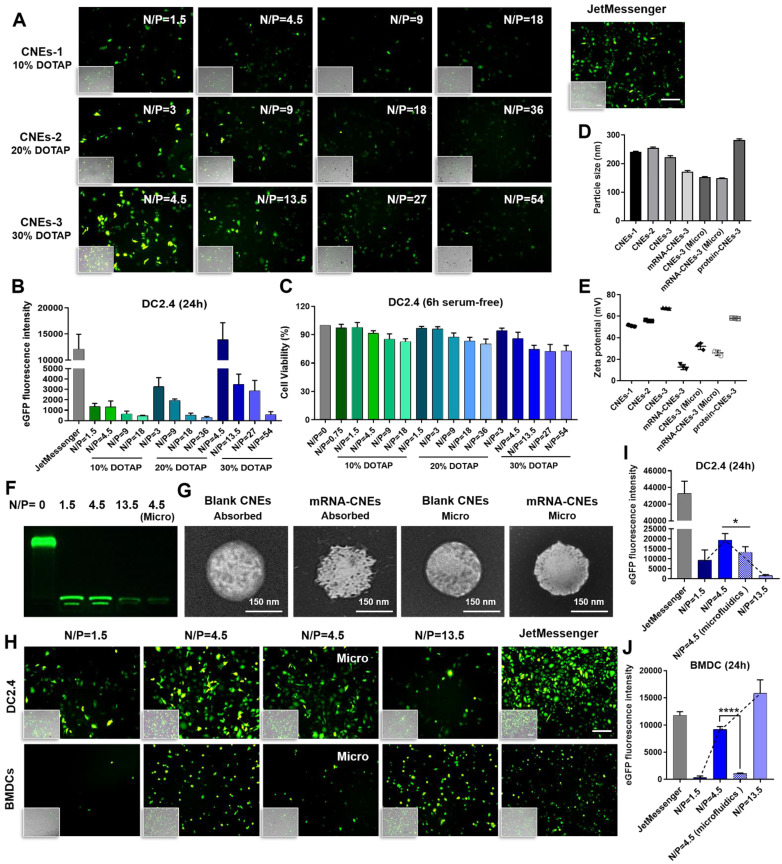
***In vitro* transfection of DCs with mRNA-CNEs complexes. (A-B)** Expression of eGFP mRNA in DC2.4 and the associated transfection efficiency at 24 h using CNEs-1 (10% DOTAP, w/w), CNEs-2 (20% DOTAP, w/w), or CNEs-3 (30% DOTAP, w/w) at varying N/P ratios (**A**). Fluorescent pictures were analyzed by Image J to semi-quantitate the fluorescence intensity of GFP protein, n = 3 (**B**). Commercial transfection reagent JetMessenger was used as positive control. Fluoresce images merged with the bright-filed vision (showing the morphology of DCs) were displayed at the bottom left corner. Scale bars, 200 µm.** (C)** Cell viability of DC2.4 treated with CNEs-1, CNEs-2, and CNEs-3 for 6 h in serum-free culture medium, n = 4. **(D-E)** Particle size (**D**) and zeta potential (**E**) of CNEs-1, CNEs-2, CNEs-3, protein-CNEs-3 (OVA protein, at a lipid/protein mass ratio of 46.7), and mRNA-CNEs complexes (eGFP mRNA, at a N/P ratio of 4.5) as prepared by electrostatic adsorption or microfluidic chip technique (Micro), n = 3. **(F)** Agarose gel electrophoresis analysis of mRNA complexation by CNEs-3. 500 ng of eGFP mRNA was incubated with CNEs-3 at a N/P ratio of 0, 1.5, 4.5 or 13.5.** (G)** Morphologies of blank CNEs-3 and eGFP mRNA-loaded CNEs-3 (prepared by electrostatic adsorption or microfluidics) under transmission electron microscope (TEM). Scale bar, 150 nm. **(H-J)** Transfection of eGFP mRNA in DC2.4 and BMDCs at 24 h with CNEs-3 at a N/P ratio of 1.5, 4.5 and 13.5 (**H**). The transfection efficiency was determined by Image J (**I-J**), n = 3. Scale bars, 100 µm. All error bars were expressed as ± SD. *p < 0.05, **p < 0.01, ***p < 0.001, ****p < 0.0001.

*In vitro* results showed that the mRNA transfection efficiency of CNEs-1 (241.5 ± 1.868 nm, 51.13 ± 0.8083 mV) in DC2.4 was not quite satisfactory (eGFP mRNA-CNEs complexes were added to serum-free culture medium for 6 h before FBS supplementation, and commercial transfection reagent JetMessenger was used as positive control, **Figure 2A-B**). Accordingly, CNEs incorporating 20% (CNEs-2, 254.9 ± 3.099 nm, 55.87 ± 0.5508 mV) and 30% (CNEs-3, 221.9.0 ± 5.82 nm, 67.6 ± 0.3606 mV) DOTAP were prepared (**Figure 2D-E**). Results suggested that the transfection efficiency was gradually improved with the increase of cationic lipid content (CNEs-3 > CNEs-2 > CNEs-1, **Figure 2A-B**), but the accompanying cytotoxicity of cationic nanocarrier 46 was also significantly elevated (**Figure 2C**), limiting the expression of mRNA at higher N/P ratios.

In order to explore the optimal transfection condition, we further investigated the expression of eGFP mRNA in DC2.4 and the hard-to-transfect BMDCs with CNEs-3 (eGFP mRNA was completely loaded by CNEs at a N/P ratio of 1.5, 4.5 and 13.5, **Figure 2F**), and found that high transfection efficiency was achieved at a N/P ratio of 4.5 for DC2.4 and 13.5 for BMDCs (**Figure 2H-J**). At the same time, CNEs-3 capable of encapsulating nucleic acids inside the nanocarrier (at a N/P ratio of 4.5) was fabricated by microfluidic chip technology (Micro, a schematic illustration was shown in **Figure S3** and described in detail in **Methods**). Transmission electron microscopy (TEM) observations suggested that both mRNA loading techniques (i.e., through post-synthetic surface adsorption and microfluidics) were able to generate well-defined nanosized complexes with spherical morphology (**Figure 2G**). In particular, the surface smoothness (**Figure 2G**) and charge (**Figure 2E**) of CNEs were significantly reduced upon mRNA adsorption, which was almost unchanged when prepared by microfluidics. We believed that the existence of cationic lipids promoted the rupture of cell membrane and facilitated the endosome/lysosome escape of cargos (proton sponge effect of cationic nanoparticles), which was conducive to the expression of mRNA. In comparison to BMDCs, DC2.4 was more sensitive to such cationic cytotoxicity, whose viability and transfection efficiency decreased sharply upon treatment (**Figure 2H-J**).

**Figure 3:**
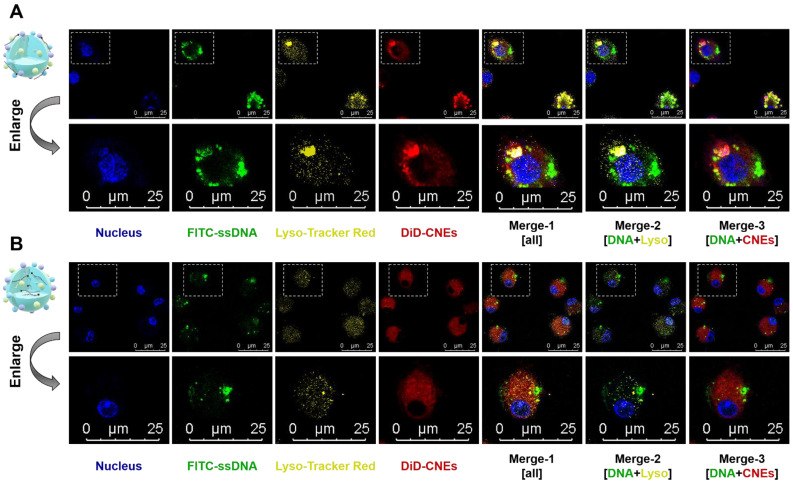
** Intracellular nucleic acid release behaviors of CNEs prepared by electrostatic adsorption and microfluidics. (A-B)** Intracellular co-localization of nucleus (blue), FITC-ssDNA (green), lysosome (yellow), and DiD-labelled CNEs (red) within BMDCs at 12 h post treatment. Cargo-loaded nanoemulsions (CNEs-3) were prepared by post-synthetic absorption (**A**) or microfluidics (**B**). Typical fields of vision were enlarged for better illustration. Scale bar, 25 µm. All error bars were expressed as ± SD, *p < 0.05, **p < 0.01, ***p < 0.005, ****p < 0.001.

It was intriguing that although mRNA-CNEs complexes produced by microfluidic technology displayed higher surface charge (**Figure 2E**), better cargo encapsulation (**Figure 2F**) and more regular shape (**Figure 2G**), its transfection efficiency was lower than that of adsorption-based one (**Figure 2H-J**). To further understand it, the intracellular nucleic acid release behaviors of CNEs prepared by electrostatic adsorption and microfluidics were investigated via CLSM. Our results showed that cargos loaded by both techniques partially released from the delivery system and avoided entering lysosome in BMDCs at 12 h post treatment (**Figure 3A-B**). In these regards, such inferior transfection ability of Micro-CNEs might result from a specific nucleic acid condensation and intracellular unpacking behavior 47, and/or caused by the different surface morphology of nanoparticles 48, 49. Based on the above results, CNEs-3 was selected to complex mRNA (at a N/P ratio of 4.5) and/or protein (at a lipid/protein mass ratio of 46.7) by post-synthetic absorption for the follow-up studies. Although nanoparticles prepared by microfluidic device were not further used in this work, such comparative studies showed that nucleic acid cargos loaded by different techniques displayed varied characteristics, which may provide some guidance for relevant researches.

### *In vitro* activation of DCs using mRNA- or protein- loaded CNEs

**Figure 4:**
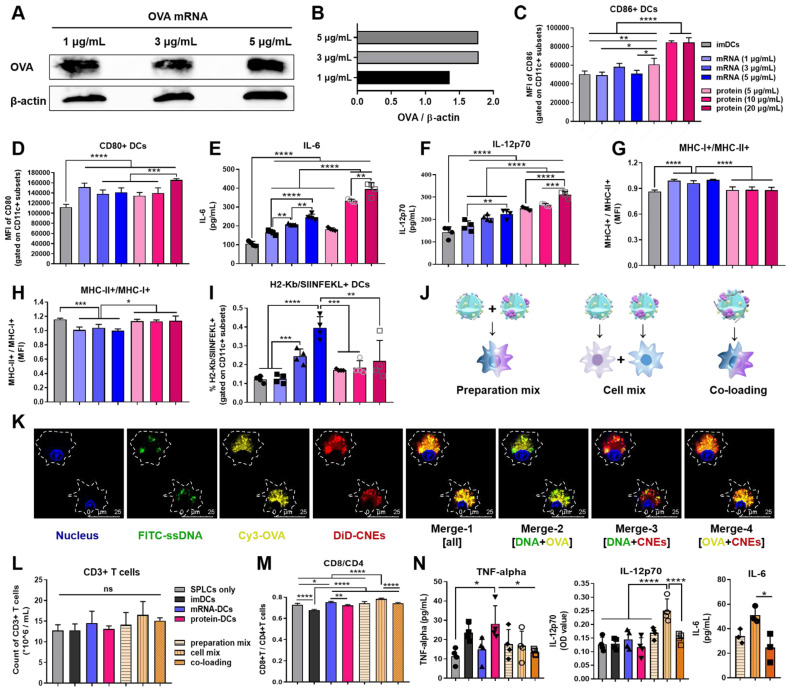
*** In vitro* maturation and T cell-activation of BMDCs treated with OVA mRNA- and/or protein- loaded CNEs. (A-B)** Expression of the OVA protein at 24 h post transfection by BMDCs using western blot analysis. OVA mRNA (1, 3, 5 µg/mL) were complexed by CNEs-3 at a N/P ratio of 4.5. β-actin was used as loading control.** (C-D)** Flow cytometric analysis of the expression of costimulatory CD86 (**C**) and CD80 (**D**) by BMDCs treated with different dose of OVA mRNA (1, 3, 5 µg/mL) or OVA protein (5, 10, 20 µg/mL) for 24 h, n = 4.** (E-F)** Determination of the secretion of IL-6 (**E**) and IL-12p70 (**F**) by BMDCs in culture supernatant by ELISA, n = 4.** (G-I)** Flow cytometric analysis of the expression of MHC-I+/MHC-II+ (**G**), MHC-II+/MHC-I+ (**H**), as well as SIINFEKL-H2K^b^ complexes (**I**) by BMDCs treated with different dose of OVA mRNA (1, 3, 5 µg/mL) or OVA protein (5, 10, 20 µg/mL) for 24 h, n = 4.** (J)** Schematic diagram of preparation mix, cell mix, and co-loading.** (K)** Intracellular co-localization of nucleus (blue), FITC-ssDNA (green), Cy3-OVA (yellow), and DiD-labelled CNEs (red) within BMDCs at 12 h post treatment. Cargo-loaded nanoemulsions (CNEs-3) were prepared by post-synthetic absorption. Scale bar, 25 µm.** (L-M)** Flow cytometric analysis of data showing the frequency of CD3^+^ T cells (**L**), and CD8^+^/CD4^+^ T cells (**M**, gated on CD3+ subsets) by OT-I lymphocytes co-incubated with activated BMDCs for 3 days, n = 4.** (N)** Cytokine profiles of TNF-alpha, IL-12p70 and IL-6 secreted by lymphocytes and BMDCs in culture supernatant using ELISA, n = 3-4. All error bars were expressed as ± SD. *p < 0.05, **p < 0.01, ***p < 0.001 and ****p < 0.0001.

Accompanied by an upregulation of costimulatory markers and cytokines secretion, the maturation of DCs is of vital importance to the initiation of immune responses 44, 50. Herein, nucleic acid and protein were complexed with CNEs-3, and different concentrations of OVA mRNA (1, 3, 5 μg/mL) and protein (5, 10, 20 μg/mL) were used to investigate the* in vitro* maturation of DCs and determine an appropriate dosage for stimulation. Western blot analysis showed a successful expression of OVA mRNA at 24 h by BMDCs (**Figure 4A-B**). And it was found that compared with immature DCs (imDCs, untreated control), mRNA- or protein- treated DCs significantly up-regulated the expression of costimulatory molecules CD86 and CD80 (**Figure 4C-D, Figure S4**), as well as the secretion of immune-potentiating interleukin-6 (IL-6) and IL-12 (dose-dependent increase, **Figure 4E-F**), suggesting that DCs were activated and matured upon antigenic stimulation. It should be noted that the cationic lipid DOTAP in CNEs might partially account for the induction of these immunostimulatory molecules (adjuvant effect), and the complement system and toll-like receptors may participate in such immune activation 51, 52. Meanwhile, mRNA induced more MHC class I-associated presentation than protein did (**Figure 4G-H, Figure S4**), including MHC-I epitope-specific (H2K^b^-restricted OVA_257-264_ (SIINFEKL)) presentation (**Figure 4I, Figure S5**). Together, these results confirmed that mRNA- and protein- based antigen incorporating multiple epitopes were able to induce both MHC class I and II immune responses, although with different preference for MHC-restricted presentation, which may contribute to a broad mobilization of the MHC system to augment anti-tumor immunity.

To investigate the T cell-activating ability of these DCs, imDCs, 3 μg/mL OVA mRNA treated DCs (mRNA-DCs), and 10 μg/mL OVA protein pulsed DCs (protein-DCs) were respectively co-incubated with OT-I mice-derived splenic lymphocytes (BMDCs:lymphocytes = 1:20) for 3 days (the TCRs of OT-I T cells can specifically recognize OVA_257-264_ in the context of H2K^b^). In addition, the T cell-activating ability of DCs matured under different mode of activation were also studied, including preparation mix ((1/5 mRNA-CNEs plus 4/5 protein-CNEs)-treated DCs); cell mix: 1/5 mRNA-DCs plus 4/5 protein-DCs, and co-loading ((1/5 mRNA plus 4/5 protein)-coloaded CNEs treated DCs) (**Figure 4J**). CLSM pictures confirmed that CNEs were capable of complexing mRNA and protein simultaneously, as the fluorescence signals of ssDNA (green), protein (yellow) and CNEs-3 (red) overlapped to a certain extent at 12 h post treatment (**Figure 4K**). Flow cytometry results indicated that different treatment conditions had little effect on the total number of lymphocytes (**Figure 4L, Figure S6**). However, imDCs and protein-DCs favored the proliferation of CD4^+^ T cells, while mRNA-DCs induced more CD8^+^ T cells (**Figure 4M, Figure S6**), which was consistent with the MHC-preference for different antigenic platforms (**Figure 4G-I**). Specifically, a simple mixture of mRNA-DCs and protein-DCs (cell mix) displayed better CD8^+^ T cell activation (**Figure 4M**) and cytokine-induction (IL-12 and IL-6, **Figure 4N**) than other two combination modes. A possible explanation to such disparity in T cell activation is that DCs treated with antigen of monotype may have higher professionality in antigen process and presentation. However, more efforts are needed to fully address this phenomenon. Taking the above results into consideration, the physical mixture of mRNA-DCs and protein-DCs was further used in the follow-up investigations.

### Optimized combination of mRNA-DCs and protein-DCs maximized anti-tumor effect

**Figure 5:**
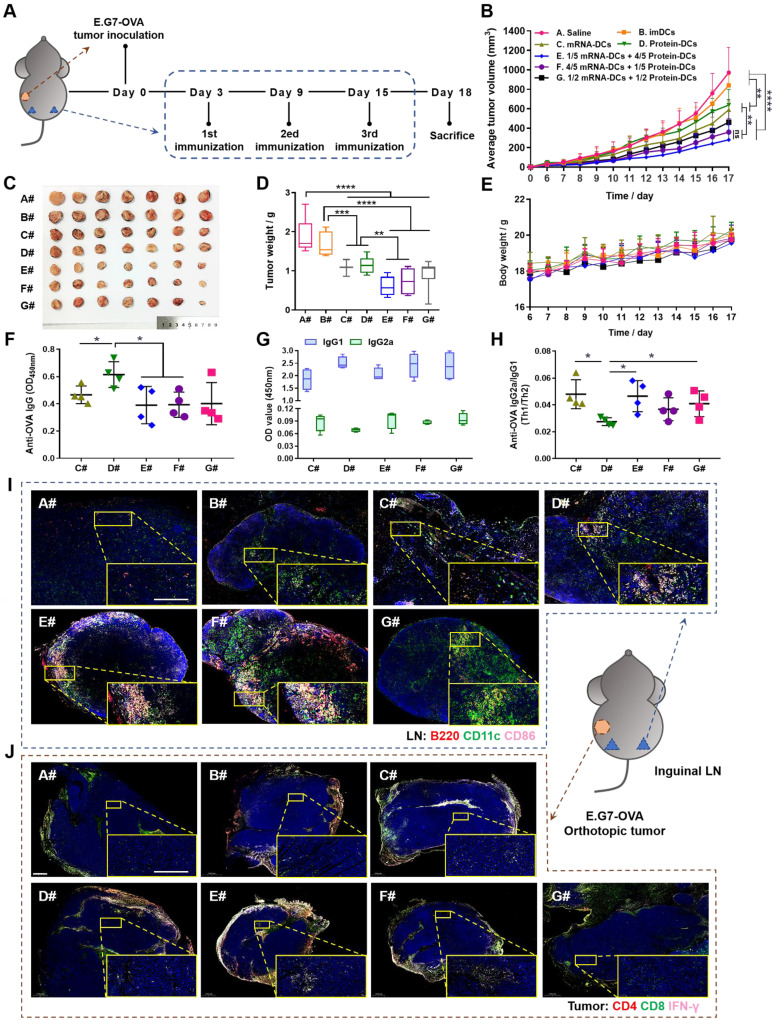
***In vivo* anti-tumor efficacy of mRNA-DCs and protein-DCs under different mode of combination against E.G7-OVA lymphoma. (A)** Schematic outline of the experimental protocol. **(B- E)** Average tumor growth curve (**B**), average body weight curve (**E**), together with pictures (**C**) and weights (**D**) of orthotopic tumors from mice in different groups, n = 7. **(F-H)** OVA-specific IgG titer (**F**), optical density of IgG2a and IgG1 isotypes (**G**), and IgG2a/IgG1 ratio (**H**) in the serum of vaccinated mice at the end of experiment, n = 4. All error bars were expressed as ± SD. *p < 0.05, **p < 0.01, ***p < 0.001, and ****p < 0.0001.** (I-J)** Whole-slide scan of immunofluorescence-stained right inguinal lymph node (LN) (**I**, blue-nucleus, red-B220, green-CD11c, pink-CD86) and orthotopic tumor (**J**, blue-nucleus, red-CD4, green-CD8, pink-IFN-γ) from each group. Typical fields of vision were enlarged. Scale bar, 200 µm.

**Figure 6:**
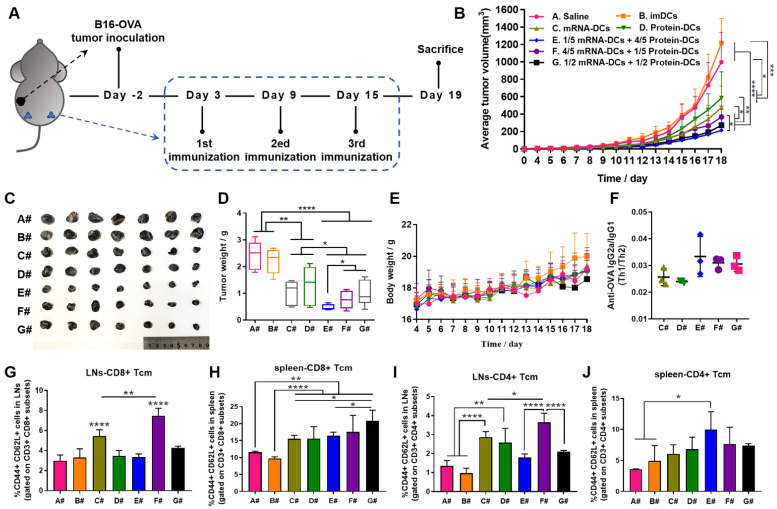
***In vivo* anti-tumor efficacy of mRNA-DCs and protein-DCs under different mode of combination against B16-OVA melanoma. (A)** Schematic overview of the therapeutic regimen.** (B-E)** Average tumor growth curve (**B**), average body weight curve (**E**), together with pictures (**C**) and weight (**D**) of orthotopic tumors of mice from different groups, n = 7. **(F)** ELISA determination of serum IgG2a/IgG1 from vaccinated mice at the end of experiment, n = 3. **(G-J)** Flow cytometric analysis of data showing the frequency of central memory (CD62L+ CD44+) CD8^+^ T cells and CD4^+^ T cells in bilateral inguinal lymph nodes (**G, I**) and spleen (**H, J**) at the end of experiment, n = 4. All error bars were expressed as ± SD. *p < 0.05, **p < 0.01, ***p < 0.001, and ****p < 0.0001.

Based on the above results, we assumed that manipulating the proportion of mRNA-DCs and protein-DCs by physical mixture before administration may affect the anti-tumor effect of DCs vaccine. Therefore, we established mouse models of E.G7-OVA lymphoma and B16-OVA melanoma, and subcutaneously vaccinated tumor-bearing mice with saline (A#), imDCs (B#), mRNA-DCs (C#), protein-DCs (D#), 1/5 mRNA-DCs plus 4/5 protein-DCs (E#), 4/5 mRNA-DCs plus 1/5 protein-DCs (F#), and 1/2 mRNA-DCs plus 1/2 protein-DCs (G#) near bilateral inguinal lymph nodes (LNs) (**Figure 5A** and** Figure 6A**). Biodistribution studies suggested that different treatment platforms had no significant effect on the *in vivo* migration of DCs, and DCs were able to migrate toward the draining LNs when subcutaneously inoculated (**Figure S7**), which might facilitate the antigenic communication between adoptive-transferred DCs and host lymphocytes, leading to an amplified immunological response.

Here, *in vivo* results from both tumor models showed that imDCs had barely any therapeutic effect, while mRNA-DCs and protein-DCs suppressed the growth of tumor to some extent, which can be further elevated when mRNA-DCs and protein-DCs were administrated concurrently (**Figure 5B-D** and** Figure 6B-D,** average body weight of mice was displayed in **Figure 5E** and** Figure 6E**). Moreover, the anti-tumor effect varied among groups treated with different combinations of mRNA-DCs and protein-DCs. This could be attributed to the fact that protein-based vaccine (protein-DCs) had superiority in inducing tumor-specific humoral immunity (as the total serum IgG titer was the highest in D#, **Figure 5F**), with cellular immunity insufficiently induced (low serum IgG2a/IgG1 ratio in D# that indicated less Th1-baised immunity, **Figure 5G-H**). The introduction of mRNA vaccine (C#) partially reversed this the situation, with serum IgG2a titer elevated in E#, F#, and G# (**Figure 5G-H, Figure 6F**). In comparison with the standalone vaccine groups (C# and D#), we observed increased frequency of CD4^+^ and CD8^+^ central memory T cells (Tcm) in the LNs and spleen from some of the combination groups (E#, F# and G#), which was different in the context of different tumor models, and the underlying mechanistic details might require more investigations (**Figure 6G-J, Figure S8-9**). Moreover, immunofluorescence staining results from E.G7-OVA lymphoma model showed that mice treated with combined DCs vaccines, especially E# and F#, displayed an increased infiltration of activated (CD86+, pink signal) B cells (B220+, red signal) and DCs (CD11c+, green signal) in the LN (**Figure 5I**), as well as an improved recruitment of functional (IFN-γ+, pink signal) CD4^+^ (red signal) T cells and CD8^+^ (green signal) T cells in the orthotopic tumor (**Figure 5J**).

**Figure 7:**
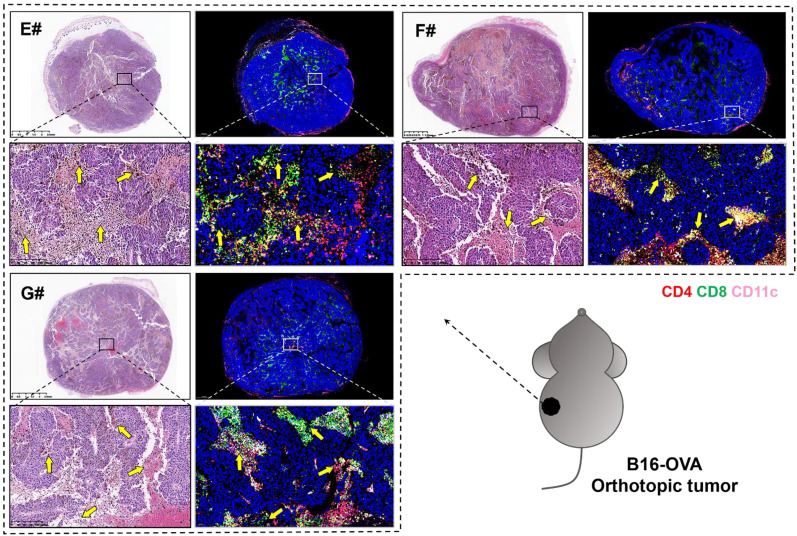
H&E (left) and immunofluorescence (right, blue-nucleus, red-CD4, green-CD8, pink-CD11c) staining of B16-OVA melanoma orthotopic tumor sections from E# (1/5 mRNA-DCs plus 4/5 protein-DCs), F# (4/5 mRNA-DCs plus 1/5 protein-DCs) and G# (1/2 mRNA-DCs plus 1/2 protein-DCs) groups. Typical fields of vision were enlarged, and necrosis areas within tumor tissues were indicated with yellow arrows.

Several studies suggested that a potent CTL response and the generation of long-lived, functional memory CD8^+^ T cells both required CD4^+^ T cell help 53, which might depend on the T cell-priming assistance, and/or the immune-potentiating cytokines and survival-associated factors provided by CD4^+^ T cells 54, 55. In order to unveil the pathological morphology and immunological landscape within tumor tissues from the combination groups, we further observed the H&E and immunofluorescence (blue-nucleus, red-CD4, green-CD8, pink-CD11c) staining of B16-OVA melanoma orthotopic tumor (**Figure 7**). There are significant tumor necrosis features in E#, F# and G# groups (indicated with yellow arrows), especially when the CD4^+^ T cells, CD8^+^ T cells and CD11c+ DCs were in close proximity to each other, suggesting that there might be certain interplay between these T-lymphocytes and antigen presenting cells (APCs) that may consequently contribute to such satisfying tumor suppression vaccine outcomes.

It's worth noting that the therapeutic disparities among E#, F#, G# in these two tumor models suggested that a personalized combination of mRNA-DCs and protein-DCs might be needed for inducing an optimum protection against different malignancies.

## Materials and methods

### Cell lines and animals

Murine B16-OVA cells, E.G7-OVA lymphoma cells (BeNa Culture Collection, Beijing, China) and immortalized dendritic cells DC2.4 (Shanghai Cell Bank, Chinese Academy of Sciences) were maintained in high-glucose DMEM medium supplemented with 10% fetal bovine serum (FBS) and 1% penicillin-streptomycin (Gibco life technologies). Mouse primary bone marrow-derived DCs (BMDCs) were generated from the bone marrow cells of C57BL/6 mouse femur and tibia, and cultured in RPMI 1640 medium containing 10% FBS, 10 ng/mL mouse recombinant interleukin-4 (IL-4, Peprotech, New Jersey, USA), and 20 ng/mL mouse recombinant granulocyte-macrophage colony stimulating factor (GM-CSF, Peprotech) for 5-6 days to obtain immature BMDCs (imDCs), as previously described 19. Cells were cultured at 37 °C in a humidified atmosphere containing 5% CO_2_ (Heraeus, Germany).

Female C57BL/6 (H-2K^b^) mice were purchased from Slaccas Experimental Animal Co., Ltd. (Shanghai, China) and OT-I mice were from Hangzhou Ziyuan Experimental Animal Technology Co., Ltd. Mice were bred under pathogen-free conditions. All experimental procedures were conducted according to the protocols approved by the Institutional Animal Care and Use Committee of Zhejiang University.

### Preparation and characterization of nanoemulsions

Oil-in-water CNEs were fabricated by a high-energy emulsification method. In brief, DOTAP, lipoid E-80 (egg lecithin-80, Avanti Co., Ltd., USA) and Vitamin E-Acetate (DL-alpha-tocopheryl acetate, BSAF) were dissolved in ethanol as the oil phase. At the same time, DEPC-treated water (DNase, RNase free) was used as the aqueous phase and added dropwise to the oil phase with vigorous stirring via vortex to produce a primary emulsion, which was further probe sonicated (30%, work 2 s, pause 3 s, 5 min, 3-4 round) on an ice bath to generate uniform nanoparticles. Prescriptions containing different content of DOTAP, or labelled with the cell membrane fluorescent probe DiD/DiR were shown in **Table 1**. Nanoemulsions (lipid concentration: 33.33 μg/mL) were all stored at 4 °C prior to use.

The morphology of CNEs was viewed by Transmission Electron Microscopy (JEOL JEM-1400 microscopes, Japan), while their particle size and zeta-potential were measured using Dynamic Light Scattering (Malvern Zeta sizer Nano-ZS instrument, UK). The cytotoxicity of CNEs to DCs in culture medium with or without serum were determined by cell counting kit-8 (CCK-8, GLPBIO, USA) assay according to manufacturer's instructions.

### Nucleic acid complexation and protein loading by CNEs

The mRNA-complexation ability of CNEs was evaluated by an agarose retardation assay. Nucleic acids were complexed to CNEs at varying nitrogen/phosphate (N/P) ratios. A total of 300 or 500 ng of mRNA was separately mixed with CNEs at different N/P ratios and allowed to complex for 30 minutes at 4 °C. Then, electrophoresis was performed with 2% (w/v) agarose gels, which were stained with Golden View™ for 15 mins at 180 V. Images were then acquired using a Bio-Rad ChemiDoxXRS system.

10 μg of OVA (MW: 44300 Da, CAS: 9006-59-1, Sigma) was dissolved in ddH_2_O and added to varying amount of CNEs, with the resulting mixture vortexed and further incubated for 30 min at 37 °C to get antigen-loaded nanoemulsions. Then, 10% SDS-PAGE coupled with fast silver stain kit (Cat No.: P0017S, Beyotime Co., Ltd) was used to investigate the protein loading ability of CNEs.

### Cell uptake and subcellular co-localization

Cells were seeded in 24-well dish with a confluence of 60-70% in 500 μL complete medium per well and treated with 3 μL FITC-OVA loaded CNEs (FITC-OVA, Solarbio Science & Technology Co., Ltd., lipid/protein mass ratio = 46.7). Here, DiD was used to label nanoemulsions, with Hoechst 33342 (10 μg/mL, Beyotime Co., Ltd.) used to visualize the nucleus. Cells were washed twice with phosphate buffer saline (PBS) and postfixed with 4% formaldehyde (10 min, at room temperature). Fluorescence images were taken with an inverted fluorescence microscope (AIR, Nikon, Japan) under constant laser intensity at 2, 4, and 8 h respectively. Images were analyzed by graphic processing software Image J to semi-quantitate the fluorescence intensity of DiD/FITC-positive cells and the number of cells. Then, different fluorescent pictures under same field of vision were merged by software EZ-MET.

BMDCs (1×10^5^ cells/well) were seeded onto 12 mm glass coverslips in 24 well plates for 16 h before transfection. For lysosomal escape assay, cells were transfected with FITC-ssDNA (1.5 μg/mL, Shanghai Generay Biotech Co., Ltd, China) -complexed CNEs or pulsed with Cy3-OVA protein (10 μg/mL) -loaded CNEs for 12 h before lysosome-staining (50 nM Lyso-Tracker Red, 30 min at 37 °C, Beyotime Biotechnology). Then, cells were fixed with 4% paraformaldehyde and post-stained with Hoechst. Finally, the slides were imaged using a confocal microscope (Leica TCS SP8, Leica, Germany). For release assay (including ssDNA-loaded CNEs (30% DOTAP, N/P = 4.5) prepared by electrostatic adsorption or Microfluidic Chip) and co-delivery assay, cells were transfected by DiD-CNEs complexed with FITC-ssDNA (1.5 μg/mL) alone or with both FITC-ssDNA and Cy3-OVA protein (10 μg/mL) for 12 h. Cells were then stained with Lyso-Tracker and Hoechst before confocal microscopic observation. Different fluorescent pictures were analyzed by software LAS X.

### Evaluation of mRNA transfection efficiency

DC2.4 (1×10^5^ cells/well) were seeded in a 24-well plate 24 h before transfection. Then, CNEs (10%, 20%, or 30% DOTAP) were incubated with 0.75 μg of enhanced GFP-encoding mRNA (5moU) (Cat#: L-7201, TriLink Biotechnologies, San Diego, CA) to reach a N/P ratio ranging from 1.5 to 54 in a total volume of 50 μL DEPC-treated water respectively for 30 min at 4 °C, while 1 μg of JetMessenger (Polyplus-transfection® SA, New York, USA) was used as positive control. Meanwhile, the transfection efficiency of mRNA-loaded CNEs (30% DOTAP, N/P = 4.5) prepared by electrostatic adsorption or Microfluidic Chip (Micronit, X3550 CH.2, Netherlands) on both BMDCs and DC2.4 was also investigated. Later, 50 μL carrier-mRNA complexes were added to each well containing 450 μL serum-free DMEM medium. Six hours later, the RPMI 1640 medium in the wells was replaced with fresh medium containing 10% FBS. Another 18 h later, cells were washed twice with PBS and imaged under a microscope, with fluorescence pictures captured and further analyzed.

### Flow cytometry, ELISA and Western Blot

Immature BMDCs were seeded in a 24-well plate and transfected with 1, 3 or 5 μg/mL of Ovalbumin-encoding mRNA (5moU) (Cat#: L-7210, TriLink Biotechnologies, San Diego, CA) that complexed by CNEs-3 (N/P ratio = 4.5), or pulsed with 5, 10, or 20 μg/mL of OVA protein (CAS: 9006-59-1, Sigma-Aldrich, USA) that loaded by CNEs-3 (lipid:protein (w/w) = 46.7). Twenty-four hours' later, the supernatant of culture medium was collected and assayed by mouse IL-12p70 (Cat#: MM-0174M1, Jiangsu Meimian industrial Co., Ltd) and IL-6 (Cat#: EK206/3, MultiSciences Biotech Co., Ltd.) ELISA kits, while cells were harvested and incubated with APC anti-mouse CD11c, FITC anti-mouse CD80, PE/Cyanine7 anti-mouse H-2K^b^ bound to SIINFEKL and PE anti-mouse H-2K^b^ (MHC class I) antibodies, or APC anti-mouse CD11c, PE anti-mouse CD86, and FITC anti-mouse I-A/I-E (MHC class II) antibodies before analyzed by flow cytometric detection (ACEA NovoCyteTM). Similarly, for OT-I mice-derived splenic lymphocytes co-incubated with BMDCs (3 days, lymphocytes:DCs = 20:1), FITC anti-mouse CD3, PE anti-mouse CD4, and APC anti-mouse CD8a were used. Antibodies used here were all from Biolegend (San Diego, USA). Data were further processed with FlowJo V10 software.

For BMDCs transfected with different dose of OVA mRNA, western blot analysis was applied to investigate the expression of OVA protein at 24 h. Briefly, cells were harvested in ice-cold PBS and lysed with RIPA buffer supplemented with protease inhibitor cocktail for whole lysate isolation. Then, proteins were electrophoresed by 10% SDS-PAGE, transferred to polyvinylidene fluoride (PVFD) membranes, and blocked with 5% bovine serum albumin (BSA) for 2 h at 37 °C. Subsequently, PVFD membranes were incubated with rabbit anti-Ovalbumin primary antibody (1:1000 diluted, 42.9 kDa, Rockland antibodies & assays) or anti-β-actin primary antibody (42 kDa, 1:1000 diluted, Proteintech) at 4°C overnight. After three hours' incubation with HRP-anti-rabbit IgG (H+L) (1:2000 diluted, Proteintech) secondary antibodies, the protein bands were detected using an enhanced chemiluminescence (ECL) system (Bio-Rad), and semi-quantitative analysis was performed with Image J.

### Biodistribution

C57BL/6 mice were subcutaneously injected with 100 μL of CNEs or PBS containing 1.2×10^6^ of naïve BMDCs, 3 μg/mL OVA mRNA- or 10 μg/mL OVA protein- pulsed BMDCs near the left inguinal LN (mRNA or protein were complexed with CNEs-3, cells were treated with preparations for 24 h before injection). Here, the biodistribution of DiR- (30 μg/mL) labelled DCs or CNEs at 12, 24, and 48 h post infusion were observed by an *in vivo* imaging system (Maestro EX, CRI Inc., Woburn, MA).

### Anti-tumor effect

To evaluate the anti-tumor efficacy of different vaccine platform-activated DCs, C57BL/6-derived BMDCs (day 6) were transfected with 3 μg/mL of OVA mRNA (mRNA-DCs) or pulsed with 10 μg/mL of OVA protein (protein-DCs) for 18 h. Then, mRNA-DCs and protein-DCs were physically mixed before adoptive transfer. C57BL/6 mice were randomly grouped (n = 7) and subcutaneously inoculated with E.G7-OVA cells (4.5×10^5^ cells/mouse) or B16-OVA (1×10^6^ cells/mouse) at the right flank. Three or five days later, first immunization was carried out, where mice from different groups were vaccinated respectively with 100 μL of saline, imDCs, mRNA-DCs, protein-DCs, 1/5 mRNA-DCs plus 4/5 protein-DCs, 4/5 mRNA-DCs plus 1/5 protein-DCs, and 1/2 mRNA-DCs plus 1/2 protein-DCs (10^6^ DCs for each mouse, s.c., near bilateral inguinal LNs). Such vaccination was performed three times at an interval of six days. Another three or four days after the last vaccination, mice were all sacrificed with serum collected and assayed for the concentration of OVA-specific IgG (mouse anti-OVA IgG1 (Cat#: 500830, Cayman Chemical), anti-OVA IgG (Cat#: 3011, Chondrex), and anti-OVA IgG2a (Cat#: 3015, Chondrex), and the orthotopic tumor and tumor-contralateral inguinal LNs were isolated for analyzing the infiltration and activation of immune cells. At the same time, their spleen and bilateral axillary LNs were harvested to investigate the memory-commitment of T cells. The body weight and tumor volume of mice were recorded every day during the experiment (tumor volume = length × width × height / 2).

### Statistical analysis

All data were evaluated and plotted using GraphPad Prism 8.0.1. Comparisons between two or several groups were analyzed using unpaired student's t-tests or one-way analysis of variance (one-way ANOVA, Tukey's multiple comparisons test), respectively. And a value of P < 0.05 was considered to be statistically significant.

## Conclusion and Discussion

In the cancer immunosurveillance hypothesis, the mechanism of immune-mediated tumor regression is restricted to different stages of tumor development 56. Aberrant activation of oncogenes drives cell into a pre-malignant state, which is carefully monitored and efficiently eliminated by CD4^+^ T cells and macrophages. However, if the immune system cannot clear pre-malignant cells in time, the latter may obtain additional genetic alterations and even transform into a malignant state where CD4^+^ and CD8^+^ T cells work together to inhibit tumor growth 24, 31. Therefore, developing anti-tumor vaccines that selectively activate CD4^+^ and CD8^+^ T cells are of vital importance.

In this study, we prepared cationic nanocarriers to promote the intracellular delivery of full-length tumor model antigen OVA in the form of mRNA and/or protein, and confirmed that DCs activated with mRNA and protein elicited MHC class I- and II- biased immunity, respectively. In exploring anti-tumor effect of DCs vaccines, mRNA-DCs and protein-DCs were physically mixed to allocate the MHC class I and II immunity for screening the best mode of combination with optimal tumor suppressing effect. Results demonstrated that a simultaneous and coordinated mobilization of MHC class I- and II- restricted responses was required for a fully effective anti-tumor immunotherapy, which might be associated with the close interplay between CD4^+^ T cells and CD8^+^ T cells.

It is worth noting that mRNA and protein encodes/incorporates multi-epitopes were used here as a tool to explore the anti-tumor effect of MHC-associated immunity, which can be further optimized by using bio-genetic engineering techniques to construct nucleic acids or protein/peptides with well-defined MHC class I or II restricted epitopes 23, 57. In addition, the expression of MHC molecules on tumor cells behaves differently not only in a variety of tumor entities, but also in tumors of similar origin 58. Therefore, characterizing the immunological properties of different tumor models is of paramount importance for determining an on-demand induction of MHC-restricted immune response by DCs vaccines. Moreover, for malignancies with low MHC expression, a coordinated mobilization of the cellular and humoral immunity, even the adaptive and innate immunity, to protect the host against a broad array of potential insults might be required. On the other hand, an orchestrated involvement of the MHC system also facilitates antiviral immunity 59, 60. Therefore, our work may also provide insights into the design and administration of future anti-virus vaccines, even boost the development of vaccines against the currently intractable severe acute respiratory syndrome coronavirus 2 (SARS-CoV-2) pandemic.

## Supplementary Material

Supplementary figures.

## Figures and Tables

**Table 1:** Formulations of CNEs-1 to CNEs-3

Lipids (mg)	CNEs-1 (10% DOTAP)	CNEs-2 (20% DOTAP)	CNEs-3 (30% DOTAP)
lipoid E-80	22.5	20	17.5
VE	22.5	20	17.5
DOTAP	5	10	15
DiD	0 (0.08)	0 (0.08)	0 (0.08)
DEPC-treated water (mL)	1.5	1.5	1.5

## Abbreviations

MHC: major histocompatibility complex
DCs: dendritic cells
CTL: cytotoxicity T lymphocyte
CNEs: cationic nanoemulsions
OVA: ovalbumin
p-MHC: peptide-MHC complexes
Th: T helper
cDC1: conventional type 1 DCs
TAAs: tumor associated antigens
TSAs: tumor-specific antigens
VE: vitamin E
PC: phosphatidylcholine
DOTAP: 1,2-dioleoyl-3-trimethylammonium-propane
BMDCs: bone marrow-derived DCs
FBS: fetal bovine serum
CLSM: confocal laser scanning microscopy
FITC-ssDNA: FITC-labelled single strand DNA
MFI: mean fluorescence intensity
TEM: transmission electron microscopy
Micro: microfluidic chip technique
imDCs: immature DCs
IL: interleukin
LN: lymph node
Tcm: central memory T cells
APCs: antigen presenting cells
GM-CSF: granulocyte-macrophage colony stimulating factor
CCK-8: cell counting kit-8
N/P: nitrogen/phosphate
PBS: phosphate buffer saline
PVFD: polyvinylidene fluoride
BSA: bovine serum albumin
ECL: enhanced chemiluminescence
SARS-CoV-2: severe acute respiratory syndrome coronavirus 2
