# Supplementary material for: Optimized mobilization of MHC class I- and II- restricted immunity by dendritic cell vaccine potentiates cancer therapy
Source: Theranostics. 2022 Apr 24;12(7):3488–502. doi: 10.7150/thno.71760 (PMC9065178; doi:10.7150/thno.71760)
Supplement: Supplementary file 1 — Supplementary figures. [file thnov12p3488s1.pdf]

Supporting information

Optimized mobilization of MHC class I- and II- restricted immunity by dendritic cell vaccine potentiates cancer therapy

Yingying Shi[#], Yu Liu[#], Jiaxin Huang, Zhenyu Luo, Xuemeng Guo, Mengshi Jiang, Xiang Li, Yichao Lu, Xu Liu, Xinyu Shan, Lihua Luo, Jian You[*]

Authors' Affiliations:
College of Pharmaceutical Sciences, Zhejiang University, 866 Yuhangtang Road, Hangzhou, Zhejiang 310058, P. R. China

*** Corresponding Author:**
Tel: 086-571-88981651
Email: youjiandoc@zju.edu.cn

[#] These authors contributed equally to this work.

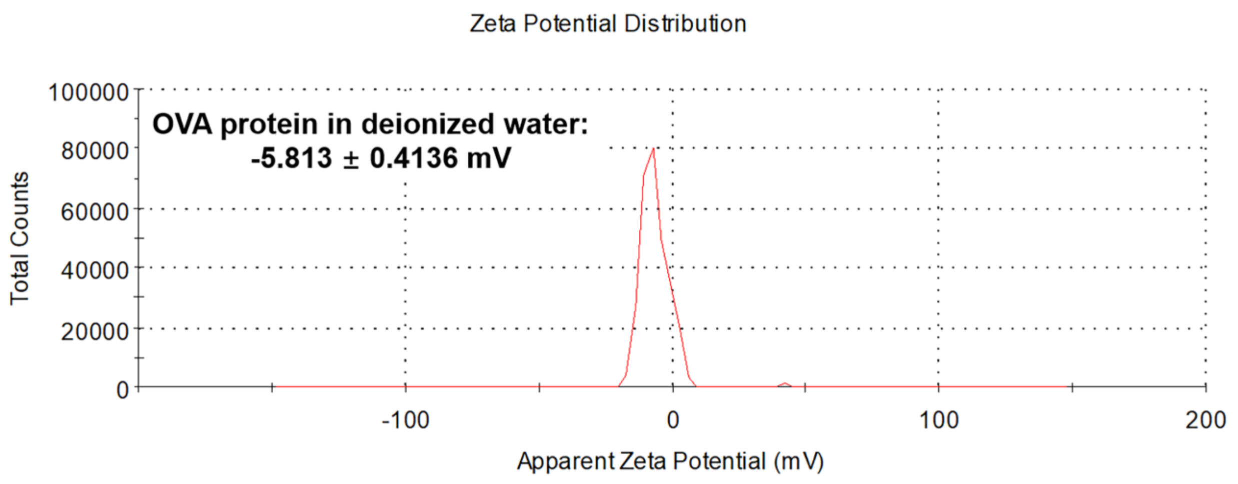

Figure S1. Zeta potential of OVA protein in deionized water.

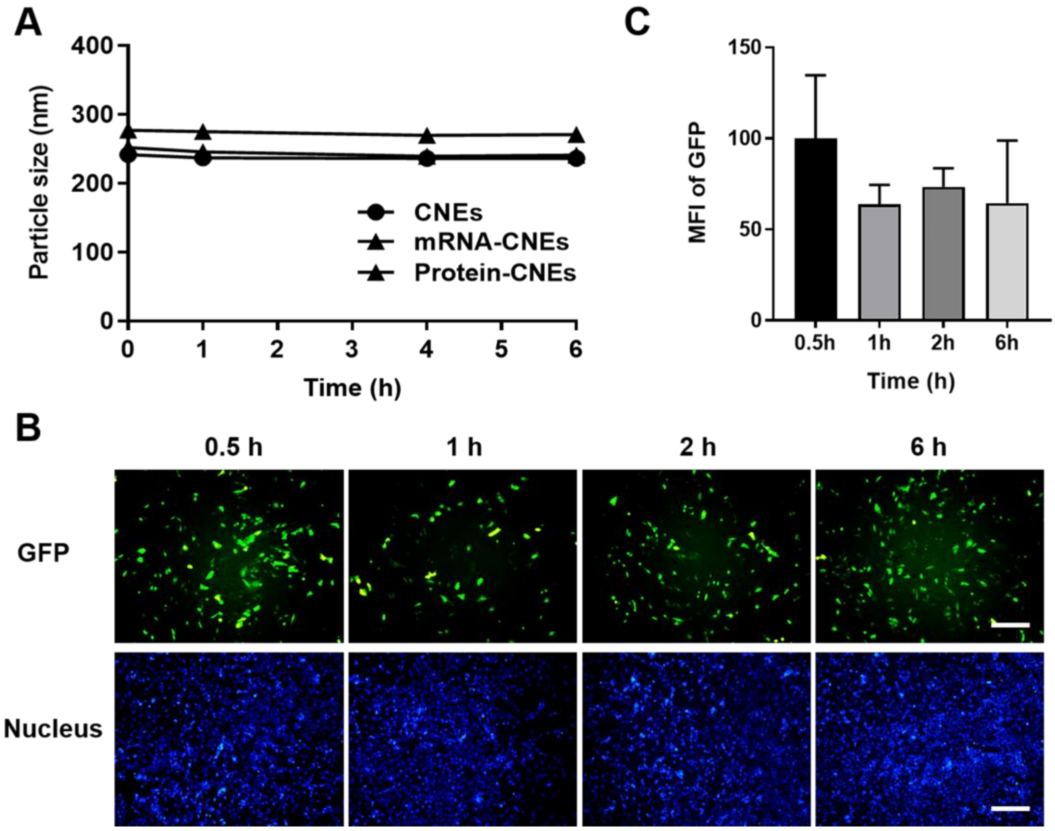

Figure S2. The stability of preparations. (A) Changes in the particle size of CNEs, mRNA-CNEs (at a N/P ratio of 4.5), and protein-CNEs (at a lipid/protein mass ratio of 46.7) within 6 h in DEPC-treated water at 4 °C, n = 3. (B-C) mRNA transfection in HEK293T cell line. The eGFP mRNA-CNEs complexes (at a N/P ratio of 4.5, 1 μg/mL eGFP mRNA) were stored in DEPC-treated water at 4 °C for 0.5, 1, 2, or 6 h before transfection (B). Fluorescence images were analyzed to determine the mean fluorescence intensity (MFI) of GFP (C), n = 4. Scale bars, 100 μm.

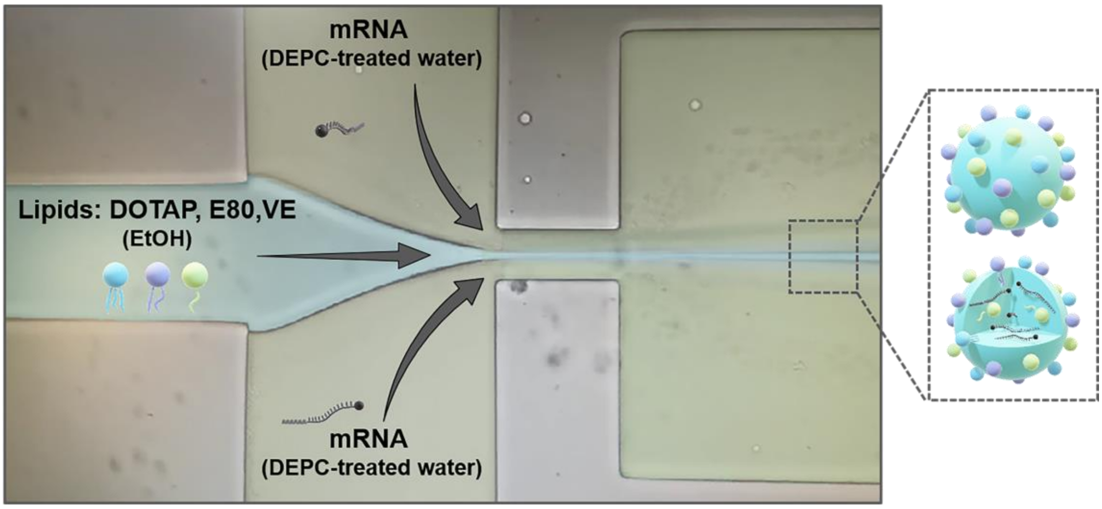

Figure S3. Schematic of mRNA-CNEs formulation process employing the microfluidic chip technology. Lipid mixture in ethanol (EtOH) and mRNA in DEPC-treated water are pumped separately into the two inlets of the microfluidic mixing device using syringe pumps a total flow rate of 10 μL/minute. Cross channel structures induce hydrodynamic flow focusing of the laminar streams to cause rapid mixing of the aqueous and ethanol phases at a flow rate ratio of 5 and correspondingly rapid increase in the polarity experienced by the lipid solution. At a critical polarity the precipitates form as LNPs. Dimensions of the mixing channel were 20 × 50 μm.

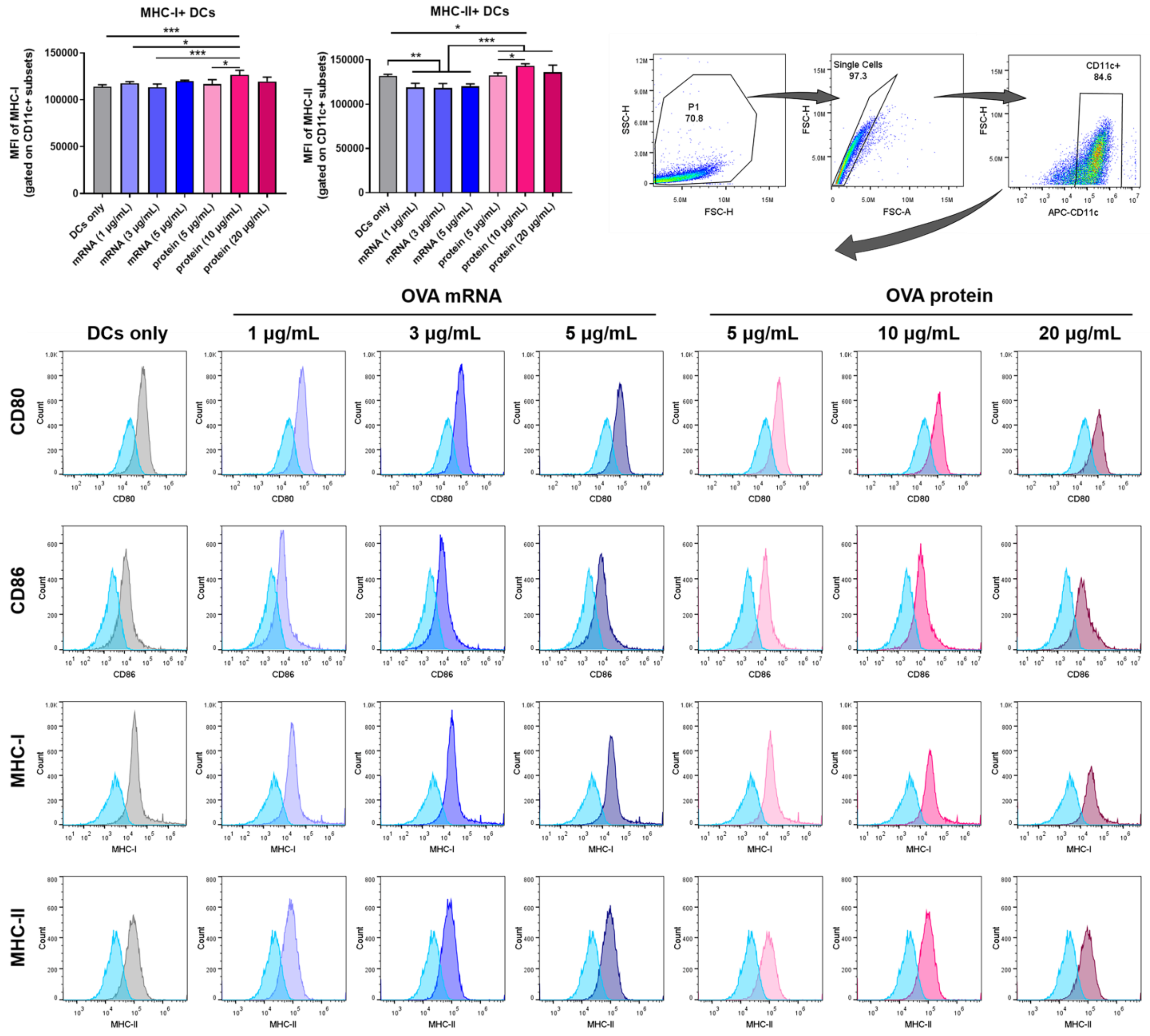

Figure S4. Flow cytometry analysis of the expression of CD80, CD86, MHC class I and II molecules by BMDCs treated with different dose of mRNA or protein *in vitro*, n = 4. The blue histogram indicates cells without staining (negative control).

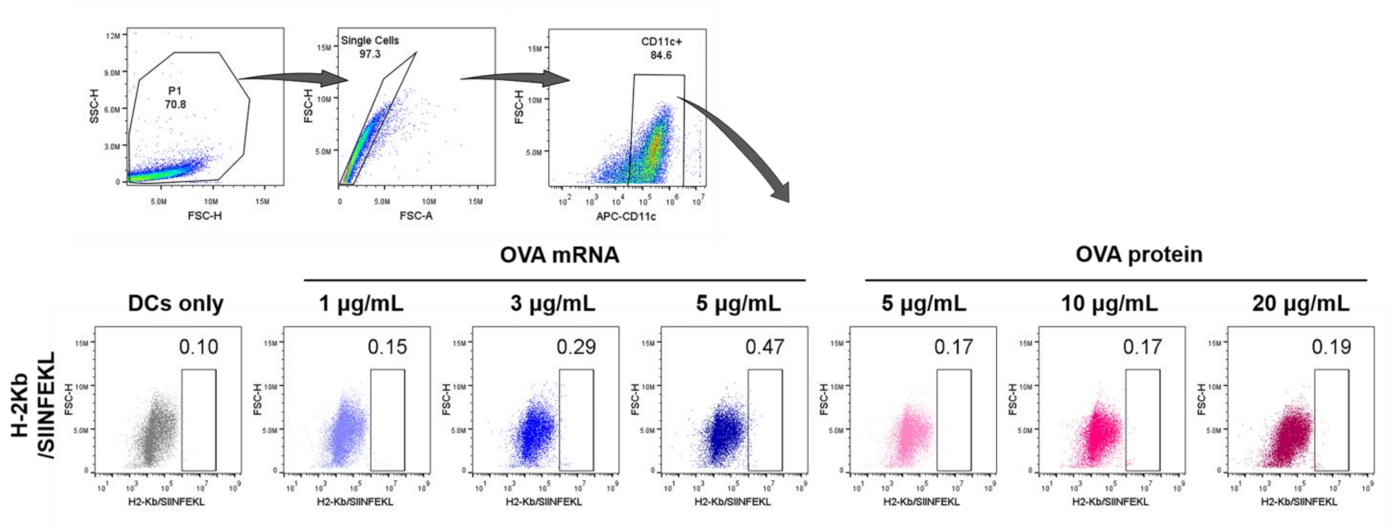

Figure S5. Flow cytometry analysis of the expression of H2K^{b}-SIINFEKL (gated on CD11c+ subsets) by BMDCs treated with different dose of mRNA or protein *in vitro*, n = 4.

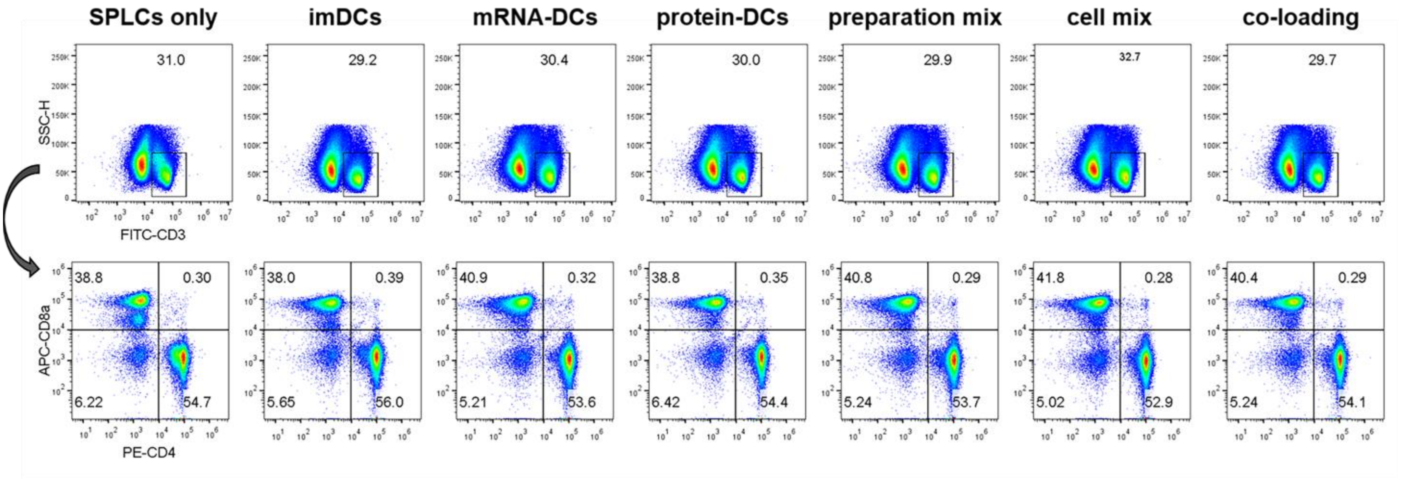

Figure S6. Representative flow cytometric pictures of data showing the frequency of CD4^{+} T cells and CD8^{+} T cells (gated on CD3+ subsets) by OT-I lymphocytes co-incubated with different preparation-treated BMDCs for 3 days *in vitro*, n = 4.

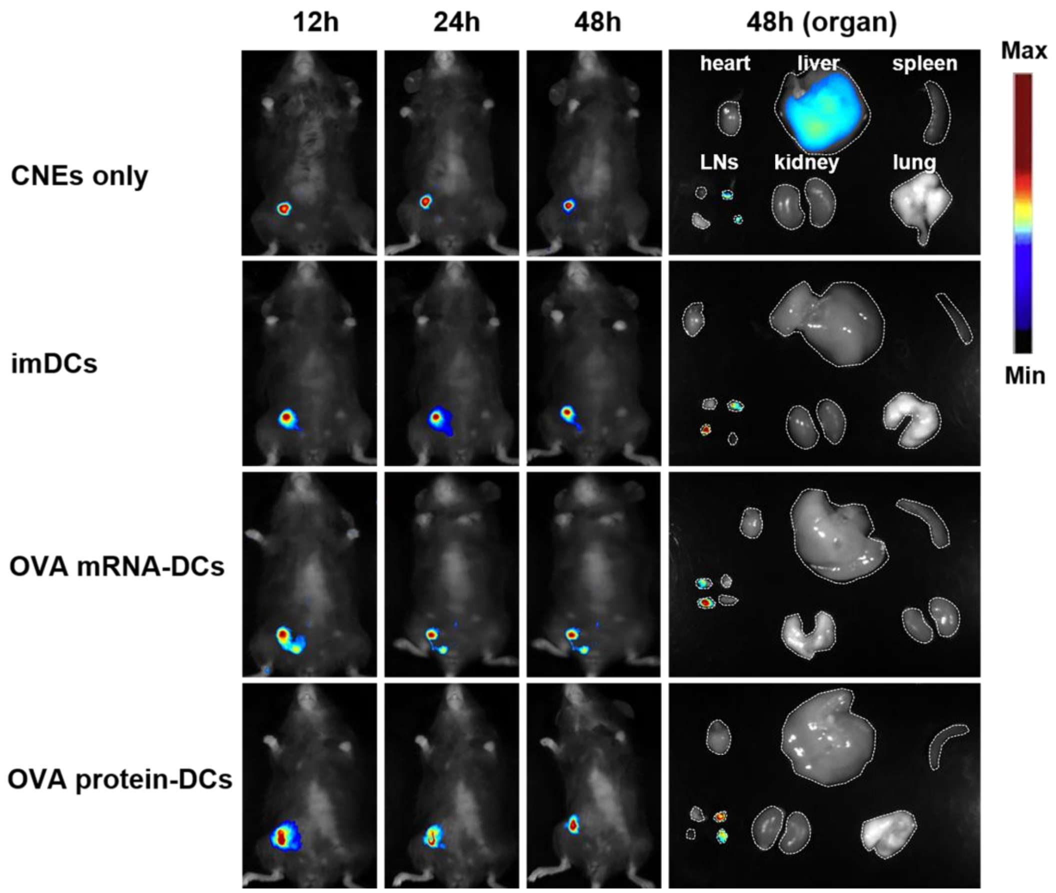

Figure S7. The biodistribution of BMDCs or CNEs at 12, 24, and 48 h post s.c. injection (near left inguinal LN). At 48 h, mice were sacrificed with major organs (heart, liver, spleen, lung, kidney, and bilateral axillary and inguinal LNs) collected for evaluating the tissue-dissemination of DCs or CNEs.

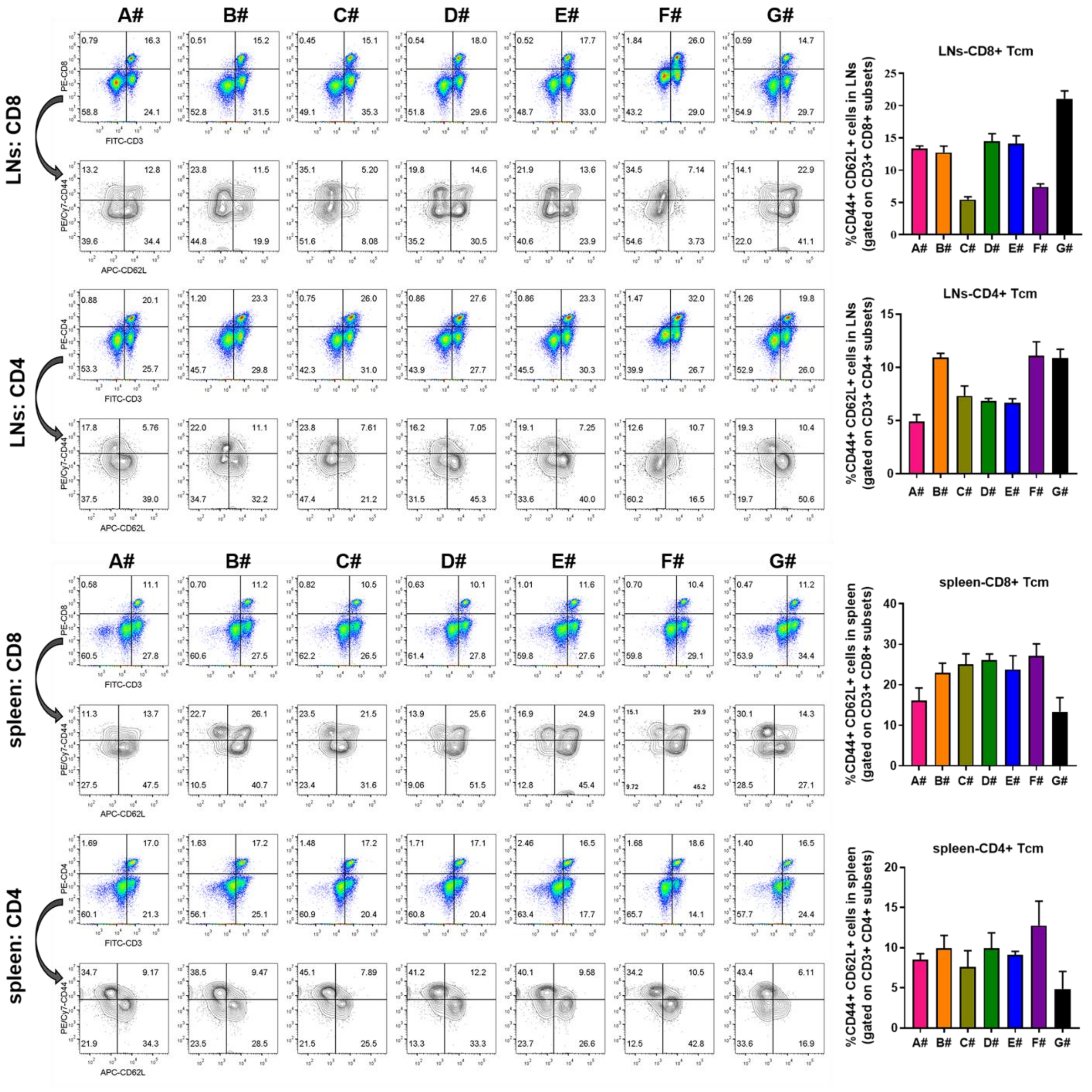

Figure S8. Representative flow cytometric pictures and summary plot of data showing the frequency of central memory ($CD62L^+$ $CD44^+$) $CD8^+$ T cells and $CD4^+$ T cells in the LNs and spleen of E.G7-OVA tumor bearing-mice from different groups at the end of experiment, n = 4.

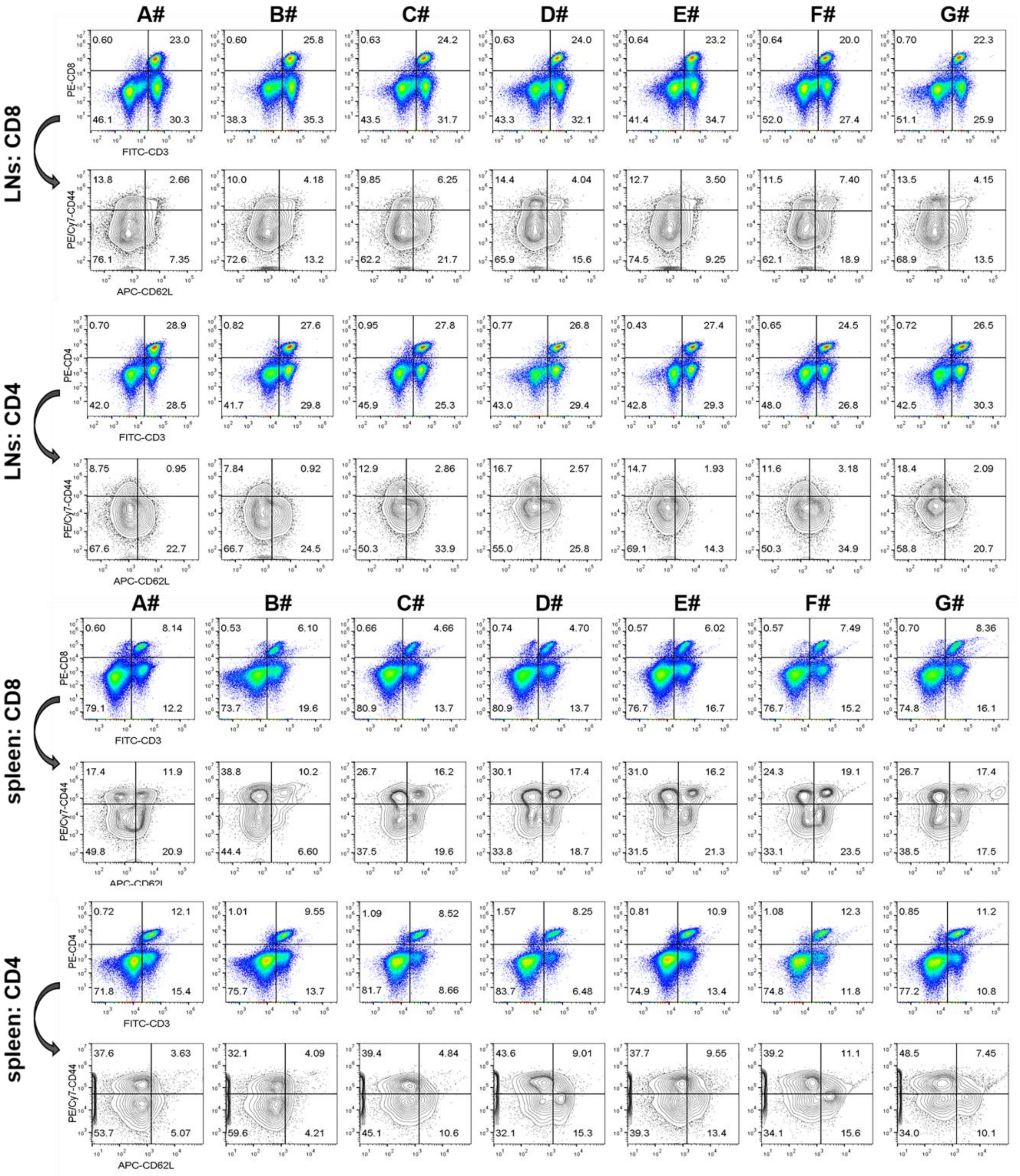

Figure S9. Representative flow cytometric pictures of data showing the frequency of central memory (CD62L$^+$ CD44$^+$) CD8$^+$ T cells and CD4$^+$ T cells in the LNs and spleen of B16-OVA tumor-bearing mice from different groups at the end of experiment, n = 4.
